# Specification of hemocyte subpopulations based on immune-related activities and the production of the agglutinin MkC1qDC in the bivalve *Modiolus kurilensis*

**DOI:** 10.1016/j.heliyon.2023.e15577

**Published:** 2023-04-20

**Authors:** Yulia Sokolnikova, Mariia Mokrina, Timur Magarlamov, Andrey Grinchenko, Vadim Kumeiko

**Affiliations:** a A.V. Zhirmunsky National Scientific Center of Marine Biology, Far Eastern Branch, Russian Academy of Sciences, 690041, Vladivostok, Russian Federation; b Laboratory of Aquacultural Biology, Graduate School of Agricultural Science, Tohoku University, 468-1 Aoba, Aramaki, Aoba-ku, Sendai, Miyagi 980-0845, Japan; c Far Eastern Federal University, 690922, Vladivostok, Russian Federation

**Keywords:** Bivalve mollusks, Cell-mediated immunity, Humoral defense, Hemocyte, Lectin-like protein

## Abstract

Bivalves, such as *Modiolus* are used as indicator organisms to monitor the state of the marine environment. Even though hemocytes are known to play a key role in the adaptive and protective mechanisms of bivalves, these cells are poorly studied in horse-mussel *Modiolus kurilensis*. In this paper, we present classification of horse-mussel hemocytes based on their immune functions, including the production of specific immune-related molecules, as well as their morphological composition after isolation by density gradient centrifugation. An effective fractionation protocol was adapted to separate four hemocyte subpopulations with distinct morphofunctional profiles. First subpopulation consisted of small under-differentiated hemoblasts (2.20 ± 0.85%) with a bromodeoxyuridine positive nucleus, and did not show any immune reactivity. Second was represented by agranulocytes (24.11 ± 2.40%), with evenly filled cytoplasm containing a well-developed protein-synthesizing apparatus, polysomes, smooth endoplasmic reticulum and mitochondria, and positively stained for myeloperoxidase, acidic proteins, glycogen and neutral polysaccharides. Third subpopulation consisted of eosinophilic granulocytes (62.64 ± 9.32%) that contained the largest number of lysosomes, peroxisomes and vesicles with contents of different density, and showed the highest phosphatase, reactive oxygen species (ROS) and phagocytic activities. Lastly, fourth group, basophilic granulocytes (14.21 ± 0.34%), are main producers of lectin-like protein MkC1qDC, recently discovered in *M. kurilensis* and characterized by pronounced antibacterial and anticancer activity. These cells characterized by intracytoplasmic of the MkC1qDC localization, forming granule-like bodies visualized with specific antibody. Both granulocytes and agranulocytes showed phagocytic activity and ROS production, and these reactions were more pronounced for eosinophilic granulocytes, suggesting that this group is the key element of the cell-mediated immune response of *M. kurilensis*. Our results support a concept of bivalve's hemocyte specification with distinct phenotypes.

## Highlights

• The hemolymph of M. kurilensis contains hemoblasts, granulocytes and agranulocytes.
• Granulocytes are the most abundant and subdivided into eosinophilic and basophilic.
• AbrHemoblasts with a BrDU-positive nucleus do not show any immune reactivity.
• Eosinophils show higher immune functions vs agranulocytes.
• Basophils are characterized by intracytoplasmic localization of MkC1qDC agglutinin.

## 1 Introduction

Bivalves are a highly heterogeneous group of organisms, that is why there is still no universal hemocytes classification applicable to all bivalves. The specialization of hemocytes in Bivalvia has been studied since the 1970s, but the particular role of hemocyte subpopulations in various reactions is still not clear, thus, not allowing to develop a unified system of their classification. This situation is affected by the lack of a unified methodological approach to the identification of hemocyte populations. Among all currently known methods, the morphological staining of hemocytes according to Romanovsky-Giemsa, May-Grunwald-Giemsa or Pappenheim for granulocytes (basophilic and eosinophilic) and agranulocytes, is recognized as widely accepted and simple, yet archaic. However, this method poorly reflects the functional features of cell populations, therefore currently it is used only as a supporting tool. Another, advanced method is the fractionation of hemolymph using cytometers and centrifugation in a density gradient of separating media. Although centrifugation has long been considered a classic, it is still used for the preparative isolation of cell populations. This method has the least effect on cell viability and activity, and also allows to compare morphological and functional data, in opposite to flow cytometry.

It is generally accepted that hemocytes of bivalves are composed of two main cell types: granulocytes and agranulocytes [1]. These two groups of cells were found in *Mya arenaria* [2], *Mytilus edulis* [3], *M. galloprovincialis* [4], *Bathymodiolus japonicus* [5], *Tridacna derasa*, *Hippopus hippopus*, *Corculum cardissa* [6], *Ruditapes decussatus* [7], *Mercenaria mercenaria* [8], *Anodonta cygnaea* [9], *Meretrix lusoria* and *Crassostrea gigas* [10], *Panopea globosa* [11], *Pinna nobilis* [12] and others. However, some authors also had found and described intermediate cell types between granulocytes and agranulocytes [[13], [14], [15]]. For example, researchers still can't agree on the existence of granulocytes in scallops, because the cells found in their hemolymph do not have the distinctive features of granulocytes, in contrast to other bivalves. Authors suggests that only a small number of semi-granular cells are present in the hemolymph, while the majority of hemocytes are represented by agranulocytes and blast-like cells [16]. In addition, granulocytes and agranulocytes are further divided into three, four or even more populations based on various parameters applied to different bivalve species. Consequently, it is often difficult to compare or generalize the results of different studies. Thus, morphologically, agranulocytes can be divided into two subclasses: small with large nuclei and large with small nuclei and large cytoplasm with a small number of cytoplasmic granules. These hemocytes, ought to perform physiological functions associated with metabolism [[17], [18], [19], [20]]. On the other hand, granular hemocytes show maximum phagocytic activity, the ability to generate ROS and produce hydrolytic enzymes that contribute to the intracellular destruction of antigens. Nowadays, the exact mechanisms of hemocyte differentiation remain unknown, but molecular methods could help to resolve this issue.

*Modiolus* is well represented genus of the family Mytilidae (over 100 species). Their ubiquitous distribution, high adaptive potential, and lack of commercial importance have made these organisms useful indicators to monitor the state of the marine environment as well as suitable models to study adaptation to dynamic processes in coastal waters [[21], [22], [23], [24], [25], [26], [27], [28], [29], [30], [31], [32], [33], [34]]. Despite the fact that hemocytes, which play a key role in the adaptation and protection of mollusks, are currently used as a main test system for assessing the physiological state of bivalves, these cells are still poorly studied in *Modiolus* [[35], [36], [37]]. Currently, no study has examined the structure, morphology, and function of *Modiolus* hemocytes, although similar classifications of hemocytes have been carried out for many members of the family Mytilidae [1]. The only paper that describes cellular composition of the hemolymph of representatives of the genus *Modiolus* is the study carried out by Anisimova [35]. In this work, three cell subpopulations in the hemolymph of *M. kurilensis* from Peter the Great Bay were identified using flow cytometry: cells with minimal granularity, cells with moderate granularity, and cells with high granularity. However, microscopic examination of the hemolymph in this work demonstrated presence of four cell populations. Currently, differences in the tools used and the criteria for cells classification do not allow a holistic view on the role of each type of hemocyte. The aim of our work was to develop a classification model of hemolymph cell populations in the bivalve *M. kurilensis*, based on the set of morphological, functional, and molecular markers, including a recently discovered protein that contains a C1q domain and targets pathogen associated molecular patterns via carbohydrate-binding activity.

## 2 Materials and methods

### 2.1 Hemolymph sampling and fractionation

Adult horse mussels (*M. kurilensis*, n = 17) were collected from the Vostok Bay of the Sea of Japan. Each mollusk was used individually for all of the subsequent experiments. Hemolymph from the posterior adductor muscle sinus was withdrawn using a 5-ml syringe with a 22G needle into a precooled 15-ml microtube to avoid hemocyte aggregation.

An aliquot of native hemolymph (10 μl) was placed on a glass slide for further morphological analysis using phase microscopy using a Zeiss Primo Star microscope (Carl Zeiss, Germany). Another aliquot (6 ml) was used for enzymatic activity analysis (paragraph 2.4). For differential hemocytes count with flow cytometer, aliquot of hemolymph (200 μl) was immediately mixed with an equal volume of 4% paraformaldehyde solution (PFA) prepared in artificial sea water (ASW) at an osmolality of 1090 mOsm and pH 7.7. The rest of the hemolymph was fractionated through isopycnic centrifugation on a discontinuous Percoll density gradient (Sigma-Aldrich, USA). Percoll was diluted in a physiological calcium and magnesium-free salt solution (CMFSS: 986 mOsm and pH 7.7) to prepare solutions with concentration of about 10, 20, 30 and 40% Percoll. An aliquot of hemolymph (4 ml) was layered over a 4 ml of discontinuous Percoll gradient and centrifuged at 800 g for 12 min at 15°С. After centrifugation, the hemocytes were layered in the interfaces between each Percoll density layer, and were collected separately using a syringe with needle, then washed three times with CMFSS with added 0.45 M ethylenediaminetetraacetic acid (EDTA), followed by centrifugation under conditions described above.

After resuspension of the hemocyte pellets in ASW solution, aliquots from each fraction were used for ultrastructural analysis and enzymatic activity analysis. The rest of the hemocytes were placed on the glass slides and incubated in a moist chamber for 20 min at 15°С for cell adhesion. Some numbers of slides were fixed for 1 h in 4% PFA solution for subsequent cytochemical analysis, staining of F-actin microfilaments, determination of cell proliferation and immunohistochemistry to detect lectin localization. Non-fixed slides were used to evaluate the lysosomal, phagocytic and ROS production activity.

### 2.2 Flow cytometry analysis

To differentiate hemocyte count, hemolymph was analyzed on a BD Accuri C6 flow cytometer (Becton Dickinson, USA) as previously described [30].

### 2.3 Ultrastructural analysis

Hemocyte suspensions from each fraction were fixed in 2.5% glutaraldehyde prepared in ASW for 1 h. Subsequently, samples were washed three times in ASW solution, embedded in 1% agarose, post-fixed in 1% osmium tetroxide for 1 h in the dark at 4 °C, washed three times again, contrasted in 1% uranyl acetate for 1 h at room temperature (RT), washed again, dehydrated using a graded series of ethanol (10 to 96%) and acetone, then embedded in mixture of Epon 812, Araldite M and DDSA (at a 1.67:1:3.67 ratio, respectively). Semi-thin sections (0.75 μm) were prepared using a HM-360 rotary microtome (MICROM International GmbH, Germany) and stained with methylene blue, then analyzed using Zeiss Primo Star microscope. Ultrathin sections (65 nm) were prepared using a Reichert Ultracut S microtome (Leica, Germany) and mounted on a copper grid using butvar film, then additionally contrasted with lead citrate. Samples were observed with a Carl Zeiss LIBRA 120 transmission electron microscope (TEM) (Carl Zeiss, Germany).

### 2.4 Enzyme detection

To evaluate enzymatic activity, native hemolymph and separated hemocyte fractions were used. All suspensions were adjusted with ASW to a final concentration of 10^7^ cells/ml as measured with hemocytometer. Hemocyte lysate was obtained with single freeze-thaw and subsequent centrifugation at 5500*g* for 10 min at 4 °C. Then, lysates were used for acid phosphatase (ACP), alkaline phosphatase (ALP) and myeloperoxidase (MPO) activity assay according to the procedure described by Xing [38]. After reaction, the absorbance was measured by a Shimadzu BioSpec-mini spectrophotometer (Shimadzu, Japan).

### 2.5 Cytochemical assays

Glass slides with fixed hemocytes were stained with different cytochemical dyes. For general morphological assay, slides were stained with May-Grunwald-Giemsa (MGG), for basic proteins detection – with fast green (pH 2.2 and 8.5), for acidic protein detection – with fast green (pH 2.2), for carbohydrate detection – with Best's carmine and periodic acid-Schiff (PAS) with Alcian blue, for lipids detection – with Sudan Black B according to the standard protocol. Then, slides were mounted in a Bio-Mount medium (Bio-Optica, Italy) and observed under a Zeiss Primo Star microscope.

Differential hemocyte count (DHC) was performed in several view fields per slide (at least 100 cells). Identification of different morphotypes of hemocytes and estimation of their percentage in hemolymph was carried out by examination of morphometric characteristics (diameter, shape, presence or absence of the studied components in the cytoplasm), nucleus staining and evaluation of cytoplasm color and its intensity.

Evaluation of the stain intensity was carried out using a semiquantitative method according to the Astaldi principle [39]. Degree of hemocyte staining was differentiated into 4 groups: negative (−), weakly positive (+), positive (++), and strongly positive (+++). The average cytochemical coefficient (CCS) was calculated using eq. (1):

(1) CCS=(A×0+B×1+C×2+D×3)

Where A is the number of hemocytes with (−) stain, B is the number of hemocytes with (+) stain, C is the number of hemocytes with (++) stain, D is the number of hemocytes with (+++) stain. Degree of staining was expressed as a scale: 0 – no stain, 0–100 – weak, 101–200 – moderate, 201–300 – strong.

### 2.6 Proliferation assay

Proliferation activity was measured by determining the incorporation of 5-bromo-2-deoxyuridine (BrdU) into hemocytes. For this, 200 μl of a 10 μM BrdU solution (MP Biomedicals, USA) was injected into the posterior adductor muscle 3 h prior hemolymph collection. Hemolymph collection and preparation of samples was carried out as described previously in section 2.1.

For DNA denaturation and permeabilization of cell membranes, fixed hemocytes were treated with 2 N HCl solution containing 0.5% Triton X-100 for 1 h at 37 °C. After hydrolysis, the prepared glass slides were treated with 0.1 M borate buffer (pH 8.5) and washed twice with 0.05 M Tris buffer (pH 7.4) with Tween-20 (TBST). To block non-specific binding of the antibodies, slides were incubated in a 3% bovine serum albumin (BSA) solution prepared with TBST for 30 min at 37 °C. The primary antibody reaction was carried out with monoclonal mouse antibodies to BrdU (Invitrogen, USA, diluted 1:100 with 1% BSA) for 12 h at 4 °C. After washing and incubation with an Alexa Fluor 488-conjugated goat anti-mouse secondary antibody (1∶400; Thermo Fisher Scientific, USA) for 2 h, slides were stained with propidium iodide solution (50 μg/ml) (PI; Thermo Fisher Scientific, USA). Then slides were embedded in Mowiol 4–88 and observed under laser scanning confocal microscope (CLSM) Zeiss LSM 700 with fluorescent filter for FITC (BP 450–490/FT 510/LP 515) and PI (BP 546/12/FT 580/LP 590) (Carl Zeiss, Germany).

### 2.7 Fluorescence staining of the F-actin

Glass slides with fixed hemocytes were incubated in 0.1% Triton X-100 for 3–5 min at RT, washed three times with ASW and stained with 100 nM rhodamine phalloidin (Thermo Fisher Scientific, USA) for 30 min in darkness at RT. Cell nucleus was stained with 4,6-diamino-2-phenylindoledihydrochloride (DAPI; Invitrоgen, USA) with a concentration of 1 μg/ml in ASW for 5 min. After three ASW washes, slides were embedded in Mowiol 4–88 and observed under CLSM with a fluorescent filter for rhodamine (BP 546/12/FT 580/LP 590). Fluorescence intensity of the F-actin was assessed in Adobe Photoshop CC 2015 program software by measuring the integral density and expressed in a procedure defined unit (p.d.u.).

### 2.8 Measurement of reactive oxygen species

To detect superoxide anions, glass slides with adhered cells were incubated in a 5 μM CellROX Orange Reagent solution (Thermo Fisher Scientific, USA) prepared with ASW for 30 min, washed three times by ASW, then fixed in 4% PFA solution for 1 h. Following cell nucleus staining step with DAPI, slides were embedded and analyzed with CLSM. During ROS oxidation in the cell cytoplasm, the CellROX had a bright orange fluorescence with excitation/absorption maxima at 545/565 nm. Fluorescence intensity was assessed in Adobe Photoshop program software by measuring the integral density and expressed in a p.d.u.

### 2.9 Phagocytosis assay

The phagocytic status of hemocytes was evaluated as previously described by Sokolnikova and colleagues [40], where the phagocytic index is the average number of bacteria ingested by one phagocyte, and the phagocytic activity is the percentage of phagocytes to the total number of hemocytes (at least 200 cells).

### 2.10 Detection of lysosomes

To detect lysosomes localization in hemocytes, acridine orange (AO; Sigma-Aldrich, USA) solution prepared with ASW (final concentration 10 μg/ml) was placed onto a slide, and covered with a coverslip with adhered living hemocytes. Then, slides were observed under a Zeiss Axio A1 fluorescence microscope (FM) with a fluorescent filter for FITC (BP 450–490/FT 510/LP 515) (Carl Zeiss, Germany). Intact AO-containing lysosomes were orange in color. The number of AO-positive cells per 200 hemocytes was counted.

### 2.11 MkC1qDC localization

Intracellular localization of the lectin-like protein MkC1qDC in hemocytes was detected immunohistochemically as previously described by Grinchenko and colleagues [41]. Fluorescence intensity was assessed in Adobe Photoshop program software by measuring the integral density and expressed in a p.d.u.

### 2.12 Data analysis

Statistical analyses of obtained numerical data were performed using Microsoft Excel 2010 and Statistica 6.0 program software. The significance differences between groups were calculated by using one-way analysis of variance (ANOVA). All data in this paper presented as the mean value ± confidence interval (95%).

## 3 Results

**Fig. 1:**
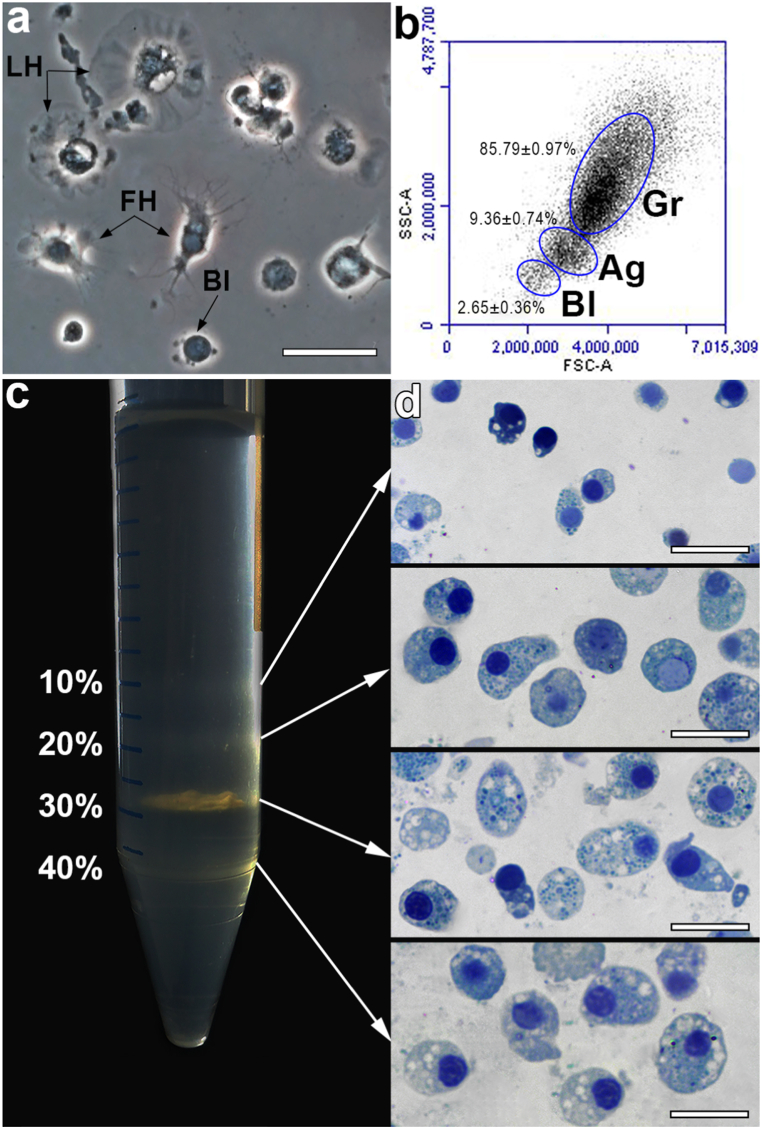
Hemocyte types and their fractions. a – phase contrast microscopy of whole hemocytes; b – flow cytometric analysis of hemolyph; c – Percoll density gradient with hemocyte subpopulations after centrifugation; d – semi-thin sections of separated hemocytes stained with methylene blue showing their morphological features. Abbreviation: LH – hemocyte with lamellipodia; FH – hemocyte with filopodia; Bl – blast-like hemocyte; Gr – granulocytes; Ag – agranulocytes. Scale bars 20 μm. (For interpretation of the references to color in this figure legend, the reader is referred to the Web version of this article.)

Observation under a microscope of the native hemolymph of the *M. kurilensis* showed that the average concentration of hemocytes was 1.77 ± 0.16 * 10^6^ cells/ml, and they are represented by several cell forms that differ in size and morphology. As shown in Fig. 1a, hemocytes with filopodia also have a body highly elevated above the surface of the substrate, while lamellipodial hemocytes spread well and occupy a much larger area of the substrate. Among hemocytes, there were also the smallest cells, that practically did not form outgrowths, but had a narrow rim of hyaline cytoplasm around the highly elevated body (blast-like cells or hemoblasts).

Flow cytometric analysis (Fig. 1b) showed the presence of three populations of cells in the hemolymph of *M. kurilensis*, differing not only in size (FSC), but also in the degree of granulation (SSC): small agranular cells or blast-like hemocytes (2.65 ± 0.36%), agranulocytes (9.36 ± 2.74%) of different sizes with minimal granularity, and granulocytes (85.79 ± 2.97%) – highly granular cells of various sizes and degrees of granularity. The content of large agranulocytes was the most variable: the concentration of these cells in the hemolymph in different animals varied from 4 to 17%. Similar phenomenon was observed regarding to hemoblasts: in some of the individuals, this form of cells was not found at all, while in others, the concentration of these cells was 8%. The minimum concentration of granulocytes did not exceed 76%, and the maximum one was about 93%.

After separation of the hemolymph in Percoll density gradient and a complex of morphofunctional examinations, four cell fractions were isolated in *M. kurilensis*. Each interface layer of Percoll contained one dominant hemocyte type in addition to small amounts of the other hemocyte types (Fig. 1 c-d, Table 1, Table 2).

**Table 1:** Cellular composition of the Percoll interfaces fractions (mean ± 95% confidence interval, predominant morphotypes and their parameters are highlighted in bold).

Fraction	Hemoblast, %	Basophilic granulocytes, %	Eosinophilic granulocytes, %	Agranulocytes, %	Cell diameter, μm	Nucleus diameter, μm
0–10%	**2.20 ± 0.85**	1.35 ± 0.22	0	1.79 ± 0.34	5.81 ± 1.54*	5.01 ± 1.36
10–20%	1.00 ± 0.10	**14.21 ± 0.34**	4.00 ± 1.21	1.33 ± 0.28	18.40 ± 4.51	4.62 ± 1.10
20–30%	0	3.72 ± 1.01	**62.64 ± 1.32**	7.24 ± 0.22	17.13 ± 7.23	4.34 ± 1.41
30–40%	0	1.44 ± 0.13	4.47 ± 2.13	**24.11 ± 2.40**	23.74 ± 3.64	4.82 ± 1.33

**Table 2:** Evaluation of the morphofunctional parameters of the hemocyte subpopulations from interfaces fractions (mean ± 95% confidence interval, predominance of the parameter is highlighted in bold). MPO, myeloperoxidase; ALP, alkaline phosphatase; ACP, acid phosphatase; PA, phagocytic activity; PI, phagocytic index; AO, acridine orange; ROS, reactive oxygen species; MkC1qDC, C1q domain-containing lectin-like protein from *M.kurilensis;* *indicates statistically significant (p < 0.05) differences.

Fraction	0–10%	10–20%	20–30%	30–40%
major cell type	hemoblasts	basophilic granulocytes	eosinophilic granulocytes	agranulocytes
Acid proteins	50	180*	91	265*
Basic proteins	10	82*	5	7
Lipids	70*	140	**207**	**255**
Acid/neutral/basic polysaccharides	0/15/0	0/86/0	**146/255/0**	**23/270/234**
MPO, U/mg protein	0.005	0.005	0.012*	**0.04**
ALP, U/mg protein	0.038	0.059	0.07*	**0.068**
ACP, U/mg protein	0.018	0.025	0.044*	0.029
BrDU-positive cells, %	2.11 ± 0.37	–	**-**	–
PA, %	3.32 ± 1.17*	17.67 ± 2.31	81.73 ± 5.41*	58.98 ± 4.76*
PI, bacteria/200 cells	3.00 ± 0.57	4.04 ± 0.94	6.21 ± 1.03	**11.05 ± 2.07**
AO-positive cells, %	–	44.11 ± 3.82*	87.13 ± 9.25*	15.00 ± 2.03
ROS, p.d.u.	3.31 ± 0.90*	15.37 ± 6.09	**30.32 ± 5.45**	**25.79 ± 5.87**
F-actin, p.d.u.	2.63 ± 0.85*	27.02 ± 4.26	36.60 ± 12.49	47.48 ± 21.36*
MkC1qDC, p.d.u.	2.03 ± 0.05*	141.16 ± 21.44*	68.45 ± 21.49	57.41 ± 28.04

**Fig. 2:**
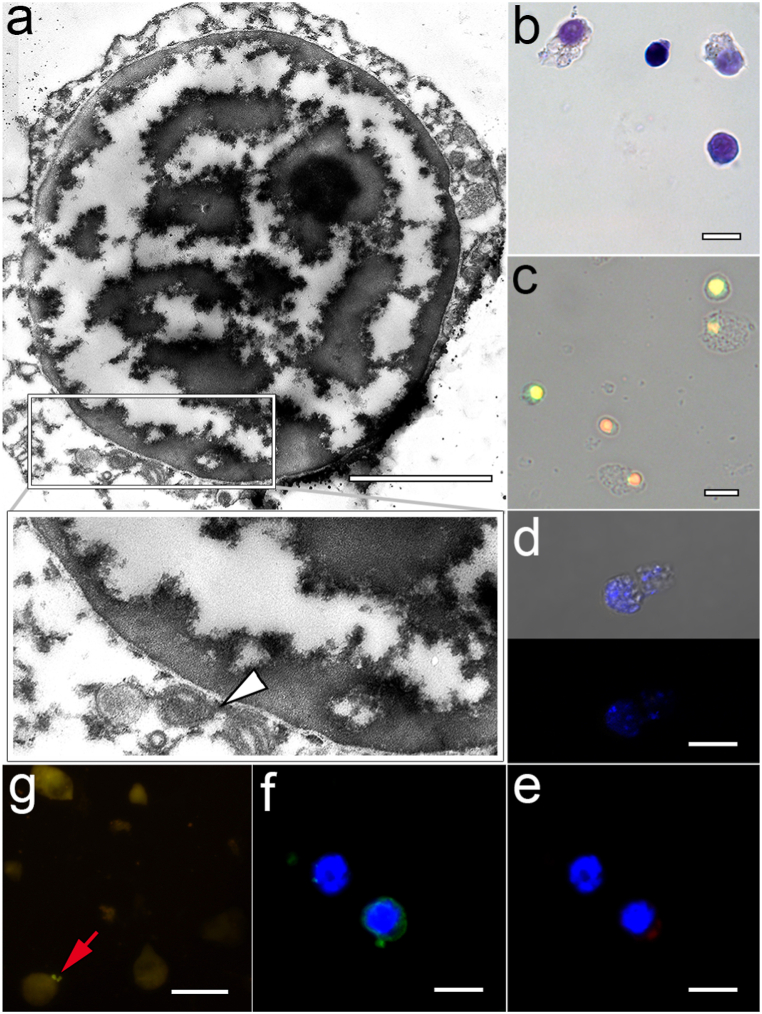
Features of hemocytes from the 0–10% fraction. a – TEM (white arrowhead – mitochondria); b – MGG staining; c – CLSM of the nuclei cells labeled with anti-BrdU Alexa Fluor-488 monoclonal antibody (green color) and PI (red color); d – CLSM of the ROS detection by CellROX Orange Reagent (DAPI-labeled nuclei in blue); e − CLSM of the F-actin microfilaments labeled with rhodamine phalloidin (DAPI-labeled nuclei in blue); f – CLSM of the MkC1qDC detection with anti-MkC1qDC Alexa Fluor-488 monoclonal antibody (DAPI-labeled nuclei in blue); g – FM of the *in vitro* phagocytosis reaction (red arrow – bacteria). Scale bars: a – 2 μm, b-f – 10 μm. (For interpretation of the references to color in this figure legend, the reader is referred to the Web version of this article.)

Hemocytes in the 0–10% Percoll fraction were the least present (Table 1). They were also the smallest (5.81 ± 1.54 μm) and under differentiated cells with a large BrdU-positive nucleus (Fig. 2 a-c) and a thin cytoplasmic rim containing nothing but mitochondria and ribosomes (Fig. 2a). Their cytoplasm gave a weak basophilic reaction (Fig. 2b), and practically was not stained with other dyes (Fig. 2 d-f). They did not show any immune reactivity (Fig. 2 f-g). This group of cells was referred by us as blast-like hemocytes/hemoblasts.

**Fig. 3:**
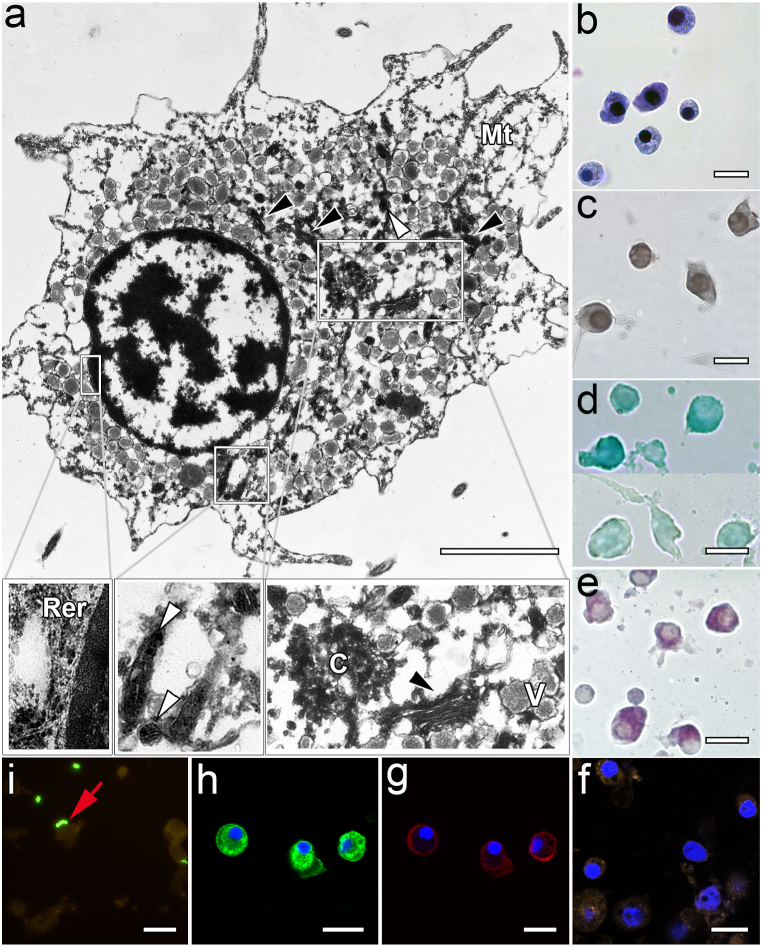
Features of hemocytes from the 10–20% fraction. a – TEM (white arrowhead – mitochondria, black arrowhead – Golgi apparatus, V – vesicles, C – centrioles, Mt – microtubules, Rer – rough endoplasmic reticulum); b – MGG staining; c – Sudan black B staining for lipids; d – fast green staining for proteins (top row – pH 2.2, bottom row – pH 8.5); e − PAS-Alcian staining for polysaccharides (purple-violet – acidic and neutral polysaccharides); f – CLSM of the ROS detection by CellROX Orange Reagent (DAPI-labeled nuclei in blue); g – CLSM of the F-actin microfilaments labeled with rhodamine phalloidin (DAPI-labeled nuclei in blue); h – CLSM of the MkC1qDC detection with anti-MkC1qDC Alexa Fluor-488 monoclonal antibody (DAPI-labeled nuclei in blue); i – FM of the *in vitro* phagocytosis reaction (red arrow – bacteria). Scale bars: a – 3 μm, b-f – 20 μm. (For interpretation of the references to color in this figure legend, the reader is referred to the Web version of this article.)

Hemocytes of the 10–20% fraction were mostly basophilic granulocytes (18.40 ± 4.51 μm) with perinuclear cytoplasm containing numerous sparsely arranged small vesicles (0.15–0.45 μm) with loose granular content, many stacks of dictyosomes of the Golgi apparatus and groups of lamellar mitochondria, that often did not exceed the size of vesicles (Fig. 3 a, b). The actin cytoskeleton was detected only in the ectoplasmic zone of the cytoplasm free from organelles. Cells of this population were the only ones whose cytoplasm, in addition to a good reaction to acidic proteins, also weakly stained for basic proteins (Fig. 3d). Staining with acridine orange for lysosomes and Sudan black B was moderately presented in this group of cells (Fig. 3c). The content of polysaccharides (Fig. 3e), actin (Fig. 3g) and ROS (Fig. 3f), as well as phagocytic activity (Fig. 3i), were insignificant in comparison with another cell fraction (20–30%) (Table 2). Whereas the content of MkC1qDC was the highest (Table 2). This specific protein was detected in numerous granule-like bodies of various sizes throughout the entire cytoplasm of hemocytes, excluding only pseudopodia (Fig. 3h).

**Fig. 4:**
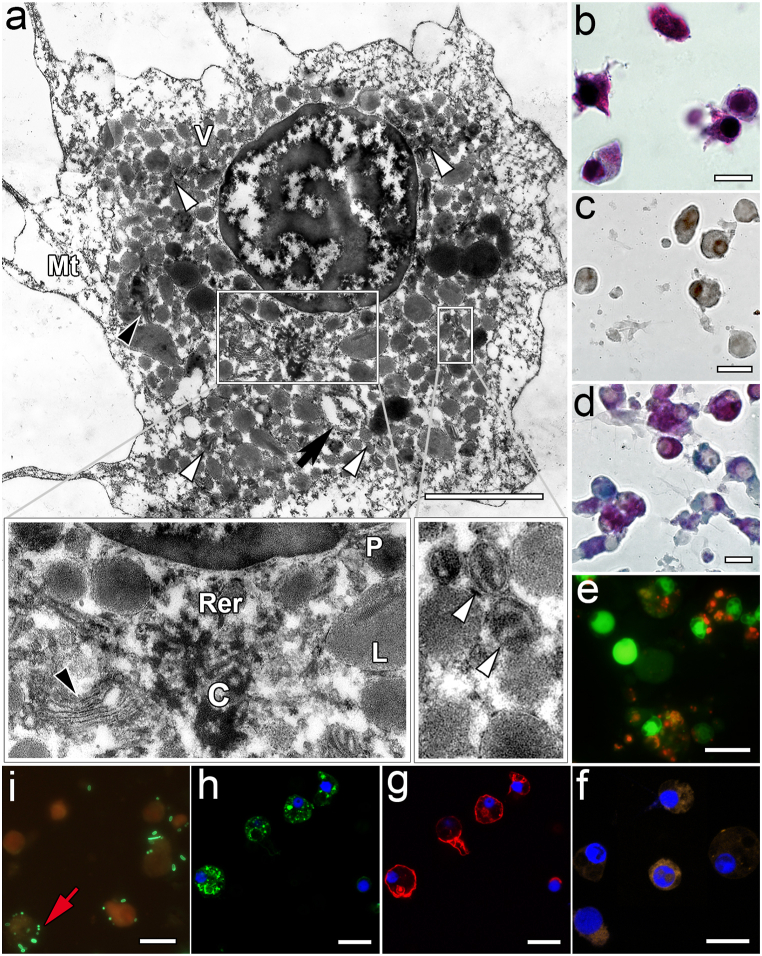
Features of hemocytes from the 20–30% fraction. a – TEM (white arrowhead – mitochondria, black arrowhead – Golgi apparatus, black arrow –residual body, V – vesicles, C – centrioles, Mt – microtubules, Rer – rough endoplasmic reticulum, P – peroxisome, L – lysosome); b – MGG staining; c – Sudan black B staining for lipids; d – PAS-Alcian staining for polysaccharides (blue – mucopolysaccharides; pink-red – glycogen and neutral polysaccharides; purple-violet – acidic and neutral polysaccharides); e − FM of the lysosomes detected by acridine orange staining (orange color); f – CLSM of the ROS detection by CellROX Orange Reagent (DAPI-labeled nuclei in blue); g – CLSM of the F-actin microfilaments labeled with rhodamine phalloidin (DAPI-labeled nuclei in blue); h – CLSM of the MkC1qDC detection with anti-MkC1qDC Alexa Fluor-488 monoclonal antibody (DAPI-labeled nuclei in blue); i – FM of the *in vitro* phagocytosis reaction (red arrow – bacteria). Scale bars: a – 3 μm, b-i – 20 μm. (For interpretation of the references to color in this figure legend, the reader is referred to the Web version of this article.)

Hemocytes from the 20–30% fraction, whose size reached 17.13 ± 7.23 μm, had granular eosinophilic cytoplasm of different density (Fig. 4b). The nuclei of these cells contained mostly euchromatin with small clumps of heterochromatin (Fig. 4a). The perinuclear cytoplasm compared to the previous cell type in the 10–20% fraction was denser (4a, c) and contained vesicles of different density and size, but smaller in number and size (0.12–0.3 μm), peroxisomes, large lysosomes (0.3–0.8 μm), numerous mitochondria and a moderately developed protein synthesis apparatus were present as well. The peripheral part of the cytoplasm was free of organelles and contained only microtubules (Fig. 4a, g). These cells were most actively involved in phagocytosis (Table 2, Fig. 4i) and showed the highest content of phosphatases (especially ACP) (Table 2), ROS, and lysosomes (Table 2, Fig. 4 e-f). This type of hemocytes stained intensely for neutral and acidic carbohydrates, but not for basic carbohydrates (Fig. 4d). MkC1qDC was detected only in some vesicles and had a looser structure in them (Fig. 4h).

**Fig. 5:**
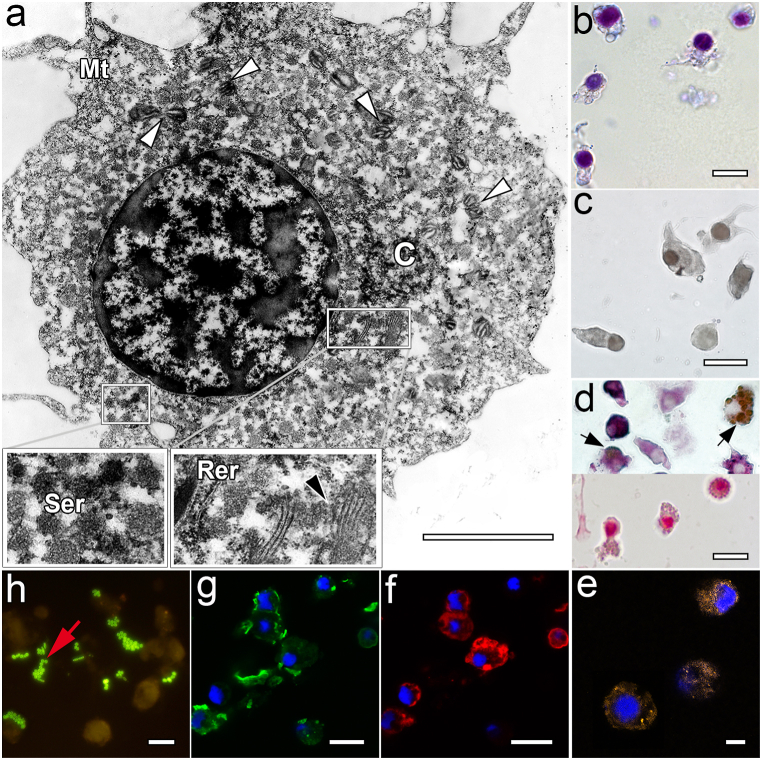
Features of hemocytes from the 30–40% fraction. a – TEM (white arrowhead – mitochondria, black arrowhead – Golgi apparatus, C – centrioles, Mt – microtubules, Ser – smooth endoplasmic reticulum, Rer – rough endoplasmic reticulum); b – MGG staining; c – Sudan black B staining for lipids; d – staining of polysaccharides (top row – PAS-Alcian, bottom row – Best's carmine, red and arrow – glycogen and neutral polysaccharides; purple-violet – acidic and neutral polysaccharides); e − CLSM of the ROS detection by CellROX Orange Reagent (DAPI-labeled nuclei in blue); f – CLSM of the F-actin microfilaments labeled with rhodamine phalloidin (DAPI-labeled nuclei in blue); g – CLSM of the MkC1qDC detection with anti-MkC1qDC Alexa Fluor-488 monoclonal antibody (DAPI-labeled nuclei in blue); h – FM of the *in vitro* phagocytosis reaction (red arrow – bacteria). Scale bars: a – 4 μm, b-h – 20 μm. (For interpretation of the references to color in this figure legend, the reader is referred to the Web version of this article.)

The last fraction (30–40%) consisted mainly of the largest (23.74 ± 3.64 μm) weakly stained cells (Fig. 5b) of various shapes – agranulocytes. No vesicles were found in their cytoplasm (Fig. 5 a-c), but it was all evenly filled with a well-developed endoplasmic reticulum, polysomes and mitochondria (greater in number and size than in the previous fraction) (Fig. 5a). Despite the fact that the cytoplasm of these cells practically was not stained with May-Grunwald (Fig. 5b), they showed the most pronounced reaction to MPO, acidic proteins, glycogen, neutral polysaccharides, and actin (Table 2, Fig. 5 d-f). The actin cytoskeleton was detected in the entire cytoplasm of the cell, excluding only a narrow perinuclear rim (Fig. 5 a, f). These cells exhibited moderate phagocytic activity, absorbing the largest number of bacteria compared to other cells (Table 2, Fig. 5 h). Another feature of this cell type was that MkC1qDC was not detected intracellularly, but only on their membrane, especially on pseudopodia (Table 2, Fig. 5g).

## 4 Discussion

In this study, hemocytes of the horse mussel *M. kurilensis* were sorted by flow cytometry and centrifugation in Percoll density gradient into three cell types: small and large agranulocytes, and granulocytes for the first time. Hemolymph separation by flow cytometry and centrifugation in density gradient showed comparable results (Fig. 1, Table 1). Granulocytes were further subdivided into eosinophilic and basophilic using morphological and functional examination. Similar groups of hemocytes have been identified using these methods in other members of the Mytilidae family [35,[42], [43], [44], [45], [46], [47], [48], [49], [50], [51]]. Like in mussel, the hemolymph of *M. kurilensis* consisted mainly of granulocytes, high-granular cells of various sizes (85.79 ± 0.97%), in accordance to the data published earlier by Anisimova [35] and Grinchenko and colleagues [37]. In above mentioned study's authors also observed prevalence in number of the granulocytes (53–63%) in the hemolymph of *M. kurilensis* from different water areas, using flow cytometry as the main method of analysis. However, as authors stated, this method was not sufficient enough for a complete characterization of hemocyte populations.

After further comprehensive examination, four subpopulations of hemocytes were identified. The first group was represented by under differentiated small hemocytes with tendency of being basophilic and a large BrdU-positive nucleus with a narrow rim of the cytoplasm. These cells did not show any immune reactivity. This group is similar to the hemocytes of *Ruditapes philippinarum* that had spindle fibers in the nucleus and were positive for anti-human CD34 antibodies that are specific to hematopoietic cells of mammals [52]. Thus, the hemocytes found by us in the upper Percoll interface fraction were classified as hemoblasts, similarly to other authors [48]. Probably, the main function of this type of hemocytes is to support other cells in circulating hemolymph during differentiation.

Another type of agranulocytes, located in the lowest interface fraction of Percoll, was represented by larger cells (24.11 ± 2.40 μm), with the cytoplasm that was practically not stained by May-Grunwald. No vesicles were found in their cytoplasm, but it was evenly filled with a well-developed protein-synthesizing apparatus, polysomes, SER, and mitochondria. Despite the fact that agranulocytes had the highest MPO activity and showed high phagocytic activity (PA), absorbing a larger number of bacteria (11.05 ± 2.07), number of acridine-labeled lysosomes and ACP activity in these cells was still significantly weaker that in eosinophilic granulocytes. Similar results regarding immune response of agranulocytes were also found in *M. galloprovincialis* [47,53] and *M. edulis* [54]. Also, in three species of *Bathymodiolus* [43], *T. crocea* [6], as well as *Cerastoderma edule* [55], agranulocytes did not show PA, that may be linked to their antigen specificity. As was reported by Takahashi and Mori [56], granulocytes of *C. gigas* exhibit high PA against three different types of bacteria and yeast cells, whereas agranulocytes showed mostly weak PA to bacteria. Analysis of the influence of certain parasites presence in the body of *M. galloprovincialis*, showed that *Marteilia refringens* causes an increase in the number of circulating agranulocytes, *Urastoma cyprinae* induces an increase in granulocytes [57]. Similarly, *Bonamia ostreae* causes an increase in agranulocytes in hemolymph of *O. edulis* [58]. Oubella and colleagues [59] reported a significant decrease in the number of agranulocytes and an increase in the proportion of granulocytes in *R. philippinarum* infected with *Vibrio tapetis*. Santarem and colleagues [60] found an increase in the concentration of agranular hemocytes in *M. galloprovincialis* upon infection with *Mytilicola intestinalis*, which again indicates the different reactivity of agranulocytes and granulocytes against various pathogens. Despite the fact that some authors [48,61,62] indicate that agranulocytes contain less ROS compared to granular hemocytes even after antigen stimulation [63], and ROS were not found at all in hemocytes of *R. decussatus*, *C. edule*, and *Corbicula japonica*, [64], we found that level of ROS production in both eosinophilic granulocytes and agranulocytes of *M. kurilensis* is almost equal. This is probably linked with high content of MPO and mitochondria that act as sources of ROS in agranulocytes compared to other types of hemocytes, which is in accordance to the work of Donaghy and colleagues [61]. In 2015, researchers proposed an experimentally confirmed mechanism of ROS production in hemocytes, according to which production of radicals occurs by cytoplasmic NADPH-oxidase, nitric oxide synthase, or MPO in stimulated cells, and by mitochondria, peroxisomes, and the endoplasmic reticulum in unstimulated cells. And since it is known that the killing of antigens can occur in oxygen-dependent and independent ways, it is likely that agranulocytes of *M. kurilensis* carry out the destruction of foreign agents mainly by ROS. However, as was shown by Terahara and colleagues [65], participation of agranulocytes in the phagocytic reaction is regulated by an integrin-dependent mechanism, in opposite to granulocytes. In addition, during spawning, there is an increase in the number of agranulocytes in the hemolymph of *M. galloprovincialis* [57], *Dreissenapolymorpha* [66] and *R. philippinarum* [67], and their decrease in *C. gigas* accompanied by a decrease in glycogen and lipid content [65]. It is found that agranulocytes are involved in wound healing [68] and in the encapsulation of the parasite *Haplosporidium nelsoni* [69] and *Tylocephalum* cestodes [70]. All these statements, paired with our data about the predominance of acidic proteins, glycogen, neutral polysaccharides and actin in agranulocytes, imply their key role in physiological processes, such as transport of nutrients and metabolites, tissue and organ regeneration [[17], [18], [19], [20]]. Thus, we assume that agranulocytes play an important role in early stages of inflammation process and migrate towards chemoattractant (most likely, damaged tissues as a result of invasion or trauma), participate in tissue clearance and formation of a connective tissue “plug” in case of trauma or capsules in case of parasitic invasion.

Despite the functional “uniqueness” of agranulocytes, it was also stated that in some mollusks they have almost the same composition of antimicrobial peptides as granulocytes, consisting of four classes: miticins, mitimycins, mytilins, and defensins [[71], [72], [73]]. In addition, basophilic and eosinophilic granular hemocytes can produce the same set of enzymes as agranulocytes, with ALP, phenol oxidase (PO), and peroxidase predominantly active [38,45]. An ultrastructural study of a number of hydrolytic enzymes localization in granular hemocytes of *M. edulis* showed the presence of arylsulfatase, glucuronidase, and elastase in large granules, while lysozyme and cathepsin B were present in all granules (however, at high dilutions, the primary antibody against lysozyme was also limited to large granules). Moreover, antibodies to cathepsin G were bounded to small granules [46]. Later, Coles and Pipe [74] reported a predominance of peroxidase activity in the small peripheral granules in eosinophils of *M. edulis* (however, not all eosinophils were positive for peroxidase). Our study revealed the predominance of lysosomes, peroxisomes, phosphatases, and ROS in eosinophilic granulocytes of *M. kurilensis*, which in combination, possibly induced their PA compared to other hemocyte types (approximately 80% of the cells of this fraction absorbed fluorescently labeled bacteria). Similar results on the key role of eosinophilic granulocytes in phagocytosis were also obtained for *M. edulis* [3], *C. edule* [55], *C. gigas* [75], *Bathymodiolus* [43] and other bivalves [73].

In addition to the above-described types of hemocytes in mussel, like Anisimova [35], we also found the presence of basophilic granulocytes in the hemolymph of *M. kurilensis*, that are smaller in size and number (14.21 ± 0.34%) compared to eosinophilic granulocytes. Based on cytometric analysis, Anisimova [35] described this type of hemocytes as semi-granulocytes – granular cells at early stage of differentiation. Our data shows that these cells contain small vesicles with electron-loose content, a large number of stacks of Golgi apparatus dictyosomes, intensely stained for acidic proteins and are the only cells that stained for basic proteins, have a low content of actin, ROS and AP, have low PA, and a moderate number of lysosomes. In literature [76,77], presence of the transitional forms, both granulocytes and agranulocytes in the hemolymph of mussel is rarely reported. Analysis of the PA of *Bathymodiolus* hemocytes showed that the most active phagocytes in these group of mollusks are eosinophils (68–74%), while basophils are less frequently involved in the phagocytosis (12–16%) [43]. Moreover, after 2 h from the beginning of phagocytosis, antigens remain intact in cytoplasm of basophilic hemocytes (even after 24 h in some cases). Basophilic granulocytes could potentially be a transitional form between basophilic hemoblasts and eosinophilic granulocytes, or still be an independent cell type, which function is still unclear. The theory of basophils and eosinophils origin from the same cell line is supported by Sekine and colleagues [5]. That work [5] shows cross-linking of group B of a monoclonal antibody (MAb Bjh4-1F4 and MAb Bjh4-3G2) with small number of both eosinophilic and basophilic granules of *B. japonicas* hemocytes. Among all the 16 MAbs they obtained, MAb Bja4-2D11, that only bounds to agranulocytes, is proved to be useful. Common epitopes between basophilic and eosinophilic granular cell types have also been described by Noel and colleagues (1994) in *M. edulis*. Carballal and colleagues [47] revealed cross-reactivity among hemocytes of different mussel species, using same antibodies: *M. edulis* MAb reacted with *M. galloprovincialis* hemocytes, and one of the MAb types showed specificity only to basophilic granulocytes of these mollusks (13B9-2E6). Currently obtained MAb libraries can also be useful not only for hemocytes classification, but also to study their functions, differentiation, and localization in the body of mollusks [78]. Thus, molecular methods have confirmed the heterogeneity of granulocytes and agranulocytes, and question of whether agranulocytes are immature cells that subsequently differentiate into granulocytes [13,48], or whether they are an independent cell line of hemocytes [45,79] was practically resolved in favor of the latest hypothesis.

Obtained MAbs for the lectin-like protein of *M. kurilensis* (MkC1qDC) by Grinchenko and colleagues in 2021 [41] showed its presence in the interstitial space and sinuses of almost all the organs tested, but was detected intracellularly only in hemocytes. A similar localization pattern has also been reported in *M. modiolus, R. philippinarum, Chlamys farreri,* and *M. trossulus* [[80], [81], [82], [83]]. It is now known that C1qDC proteins in bivalve mollusks can be expressed in all organs and tissues, but with varying degrees, which increases after exposure to various microorganisms [[84], [85], [86], [87], [88]], or their components associated with pathogen-associated molecular patterns (PAMPs) [88,89]. Main functions of lectin-like proteins in bivalves are agglutinating, cytotoxic, and bacteriostatic [87,[90], [91], [92], [93], [94]]. However, roles other than immune response have been suggested in some organs [95], such as food sorting regulation and cell-to-cell interactions [[96], [97], [98], [99], [100]]. In our previous study, the MkC1qDC lectin from *M. kurilensis* had a unique range of carbohydrate affinity, exhibited antibacterial activity against gram-negative and gram-positive strains and interactions with PAMPs (mannan, LPS, PDG), and dose-dependently suppressed the proliferation of HeLa cells of human adenocarcinoma [41]. Immunolabeling of different types of hemocytes with MAb for MkC1qDC showed differences in its localization: lectin was not detected at all in blast-like cells, only on the cell membrane in agranulocytes, and in granules of granulocytes. The highest concentration was observed in basophilic granulocytes. Membrane localization of lectin in agranulocytes may be associated with the implementation of their regulatory functions, in which the protein acts as a transmitter molecule.

Tasumi and colleagues [101] examined the subcellular localization of the mature CvGal protein in both circulating and adherent hemocytes in *C. virginica*, and it was found that CvGal is present in the cytoplasm of one third of circulating hemocytes. During attachment and spreading, the lectin moved to the periphery of cell and was secreted into the extracellular space [101]. CvGal was secreted by attached granulocytes, bound to the cell surface, and the remaining galectin was released into the environment. The soluble CvGal then bound to all other circulating (non-activated) cells (both granulocytes and hyalinocytes/agranulocytes). Although hyalinocytes may lack synthesis mechanisms for CvGal, they express cell surface integrins, which are well known as galectin ligands [102]. Thus, the binding of CvGal to hyalinocytes can lead to their phagocytic activation. Our data, combined with literature, indicate that lectins can be useful for hemocyte types differentiating. In future, observation of the hemocyte types labeled with lectin under short-term cell culture condition, could potentially contribute to the understanding of the histogenetic relationships of hemolymph cells not only in *M. kurilensis*, but in bivalves in general.

Our study confirmed that granulocytes and agranulocytes are the main circulating hemocytes of *M. kurilensis* and they share morphological and functional characteristics with hemocytes of other bivalve species. Thus, we suggest that ultrastructure of cells, MGG staining, enzymatic composition, PA, lysosomal activity, content of acidic proteins and lipids are most valuable parameters for hemocytes classification. Proposed cell classification algorithm based on Percoll fractionation can be recommended as an optimal method for morphofunctional classification of bivalve hemocytes with minimal resources. Also, lectins mentioned in this work can also be used in future as an addition to the description and classification of hemocytes, and thereby increase quality of existing cell classification models.

Further study of hemocytes subpopulations of *M. kurilensis* in combination with transcriptomic analysis will allow more detailed characterization of the functional differentiation of hemocytes. For example, in the work of Meng and colleagues [103], several granulocyte subpopulations were identified in hemolyph of *R. philippinarum* and *C. hongkongensis* by transcriptomic analysis. The only work that combined transcriptomic and morphofunctional analysis of individual cell populations obtained by Percoll density gradient centrifugation was performed by Wang and colleagues [75]. In that study [75] the differential expression of the immune-related genes (CgTLR, CgClathrin, CgATPeV, CgLysozyme, 24 CgDefensin, CgIL-17) was observed both in agranulocytes and granulocytes, and was more intense in granulocytes. Also, Mao and colleagues in 2020 [104] revealed a set of differentially expressed genes of sorted granulocytes and agranulocytes of *C. gigas* using quantitative transcriptomic analysis. These genes were predominantly expressed in granulocytes, confirming the heterogeneity of these two groups of hemolymph cells.

Our data support a concept of molluscan hemocyte specification with distinct phenotypes of eosinophilic granular hemocytes responsible for cell-mediated immunity and basophilic granular hemocytes that actively involved in humoral immune response via specific factor production (MkC1qDC) targeting PAMPs. Further studies on gene expression profiles in combination with experimentally induced MkC1qDC biosynthesis could help us to understand better the hemocyte response, serving for diagnostics of the molluscan health in normal or pathogenic environment.

## Author contribution statement

Yulia Sokolnikova: Conceived and designed the experiments; Analyzed and interpreted the data, Wrote the paper.

Mariia Mokrina: Performed the experiments; Analyzed and interpreted the data.

Timur Magarlamov, Andrey Grinchenko: Performed the experiments.

Vadim Kumeiko: Contributed reagents, materials, analysis tools or data; Wrote the paper.

## Funding statement

This work was supported by Ministry of Science and Higher Education of the Russian Federation [FZNS-2023-0017].

## Data availability statement

Data included in article/supp. material/referenced in article.

## Declaration of interest’s statement

The authors declare no competing interests.
